# An Evaluation of the Timing and Use of Healthcare during Pregnancy in Birmingham, UK and Pretoria, South Africa

**DOI:** 10.5402/2011/364243

**Published:** 2011-01-26

**Authors:** Mark Robert Openshaw, Hlwelekazi N. Bomela, Sam Pretlove

**Affiliations:** ^1^University Hospital Birmingham NHS Foundation Trust, Queen Elizabeth Medical Centre, Edgbaston, West Midlands, Birmingham B15 2PR, UK; ^2^Department of Paediatrics (Neonatology) Steve Biko Academic Hospital, University of Pretoria, P.O. Box 667 Malherbe Street, Capital Park, Pretoria 0001, South Africa; ^3^Department of Fetal and Maternal Medicine, Birmingham Women's Hospital NHS Foundation Trust, Metchley Park Road, Edgbaston, Birmingham B15 2TG, UK

## Abstract

*Objective*. A pilot study to compare the rates of antenatal healthcare use in Birmingham, UK and Pretoria, South Africa, and identify differences in knowledge and perception of antenatal healthcare. *Subjects*. 62 women, 31 at each location <24 hours after delivery. *Results*. Women from Birmingham use healthcare services earlier (*P* ≤ .0001) and more often during pregnancy (*P* ≤ .0001). Women from Birmingham identified more conditions that may affect pregnancy (median 6 versus 3 reasons) and were less aware of HIV. In addition they perceived antenatal healthcare as relatively more important for advice and reassurance about pregnancy, whilst women from Pretoria had more problems with transport and clinic overcrowding. *Conclusions*. Increasing education on the importance of antenatal healthcare and medical problems during pregnancy may help improve antenatal healthcare use in Pretoria. Improving transport links and overcrowding in clinics in Pretoria may also help increase use. Measuring maternal outcomes and confirming these findings in a larger population are important for future studies.

## 1. Introduction

Antenatal care is believed to have a positive impact on pregnancy outcomes, either through early diagnosis and intervention for complications, or by contributing to the elimination and reduction of modifiable maternal risk factors. The recommended antenatal care programme in less developed countries is often the same as the programmes used in developed countries [1]. However, across the world there is wide variation in the proportion of women who receive antenatal care. Women differ in the access they have to antenatal healthcare, the perceived need for it, and the use they make of it [2].

Most women in the United Kingdom (UK) are aware that there are many health risks to themselves and the unborn child, and that there are many tests that may detect these abnormalities. For these reasons less than 1% of British pregnant women have no antenatal tests [3]. Most women make first contact with dedicated antenatal healthcare services between the 11th and 13th weeks, and NICE guidelines recommend seven–ten appointments [4]. The guidelines for South Africa are for a minimum of one visit in each trimester [5].

In contrast to the UK where comprehensive, free antenatal care began in 1948, South Africa only began offering universal antenatal care in 1994 [6]. Despite the widespread availability of antenatal healthcare most women in rural South Africa attend their first antenatal clinic in late pregnancy [7] at an average of up to 25 weeks [8]. Inadequate antenatal care, due to late booking and failure to follow up, contributes to large numbers of avoidable perinatal deaths and maternal complications, and is one of the avoidable factors underlying maternal mortality [8, 9]. Lack of easy physical access to healthcare and difficulty in recognizing pregnancy as well as educational factors have been identified as reasons for late use of antenatal healthcare by Hamilton et al. [10].

As the reasons for these differences in healthcare attendance are poorly understood, we developed an interview based on a structured questionnaire to measure antenatal healthcare use and identify important differences in knowledge and perceptions of antenatal healthcare that may account for differences in use. (See Questionnaire in Supplementary Material available online at doi: 10.5402/2011/36243.)

## 2. Material and Methods

We designed a comparative cross-sectional pilot study of postnatal women in two populations; one at Birmingham women's hospital in the UK and the other in Pretoria academic hospital (now renamed Steve Biko academic hospital) in South Africa. Birmingham women's hospital is a state-run hospital for women in the West Midlands region of the UK primarily providing antenatal care for south Birmingham. Pretoria academic hospital is a state-run teaching hospital in the Tshwane district of Pretoria. The Pretoria data was collected 01–11/05/2007 and the Birmingham data was collected 25–29/06/2007. Thirty-one patients were interviewed at each location, this being the maximum number that could be interviewed in the 10-day period in Pretoria. Three women from Birmingham and five from Pretoria declined interview.

Interviews were conducted on women whom had given birth to healthy babies within the last 24 hours (both normal vaginal delivery and caesarean section). Sampling was systematic where all patients who fell within the target population in the maternity wards on consecutive days were interviewed. Following approval by the south Birmingham ethics committee and the ethics committee of Pretoria academic hospital and trust approval and indemnity from Birmingham women's hospital, structured questionnaires were completed as bedside interviews, by the main author, next to mother and baby. 

Information was collected on sociodemographic details; timing and use of antenatal healthcare; reasons for and barriers to the use of antenatal healthcare. Data was a mixture of categorical sociodemographic data, quantitative variables on antenatal healthcare, use and qualitative comments. Nurses acted as interpreters on the ward for women who could not speak English. All variables were analysed using Mann-Whitney tests in Minitab 15.

## 3. Results

### 3.1. Response and Sociodemographics

Thirty-six patients from Pretoria and thirty-four patients from Birmingham were approached for interview of which and five and three declined, respectively. A total of thirty-one patients were interviewed at each location. In Birmingham, the interview population was 64% Caucasian, 23% Asian, 10% Afro-Caribbean, and 3% “other” with 2 Asian and 1 Caucasian woman declining interview. Of those interviewed in Pretoria 100% were black South Africans. Two white South Africans, two black South Africans, and one Asian woman declined interview. In both populations all patients gave an urban address. The Birmingham population had a median monthly income of *£*1500 (range *£*200–*£*15,000) whilst the Pretoria median monthly income was 1200 Rand (~*£*920) (range 140–2600 Rand) (*P* = .0001). The employment rate was 58% versus 39% for Birmingham and Pretorian women, respectively (*P* = .1325). 

**Figure 1:**
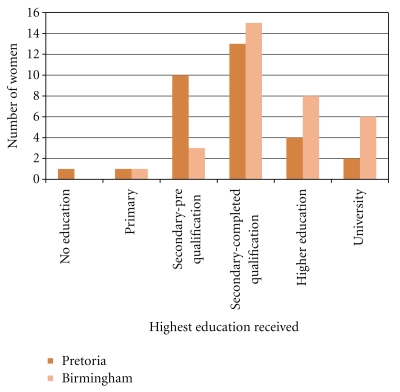
Highest level of education achieved by women from Pretoria and Birmingham.

Women from Birmingham had their first pregnancy at a median of 24 years (range 17–40) and women from Pretoria at a median of 21 years (range 15–28). Birmingham women were significantly older at first pregnancy (*P* = .0027), however there was no significant difference in terms of age at interview or in number of pregnancies or live births. Women from Birmingham received a higher level of education than those in Pretoria (Figure 1).

### 3.2. Healthcare Use and Awareness

**Figure 2:**
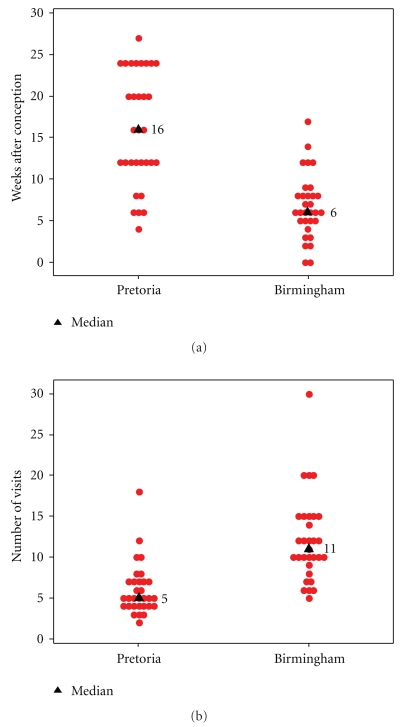
Scatter-plots showing the time of first visit to a healthcare professional (a) and number of visits made (b) by pregnant women in Pretoria and Birmingham.

Women in Birmingham attended antenatal clinics earlier (*P* < .0001) in pregnancy, median 6 weeks after conception (range 0–17), than those from Pretoria, median 16 weeks (range 4–27). Women in Birmingham also had more frequent visits (*P* < .0001) than those from Pretoria: median 11 visits (range 5–30) during pregnancy in Birmingham; median 5 visits (range 2–18) in Pretoria. (Figures 2(a) and 2(b)). 

**Figure 3:**
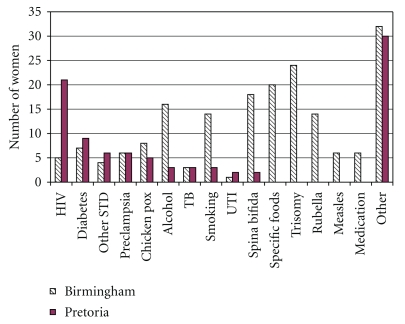
Conditions identified by women that may affect pregnancy.

The two populations differ markedly in their knowledge of the problems that may occur during pregnancy. Women from Birmingham could name more conditions that may affect pregnancy than those from Pretoria with a median of 6 reasons (range 1–10) versus 3 reasons (range 0–8), respectively (*P* ≤ .0001). Women from Pretoria were more aware of HIV as a problem (Figure 3). 

### 3.3. Reason for Seeking and Barriers to Healthcare

Women from both regions stated that checking the health of the baby and their own health were the two most important reasons for seeking antenatal healthcare (Table 1). Some women from Pretoria felt that discovering their HIV status was important (9.7%), whereas none from Birmingham felt likewise. Indicating that South African women are more enlightened about the HIV infection and may seek antenatal care to check their HIV status. Furthermore, women from Birmingham placed much more emphasis than women from Pretoria on “advice” (35.5% versus 6.5%) and “reassurance” (16.1% versus 3.2%) as a function of antenatal healthcare. 

**Figure 4:**
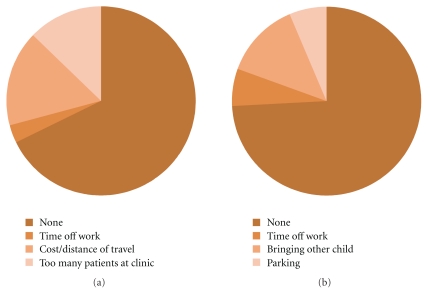
Barriers to antenatal healthcare identified by women from Pretoria (a) and Birmingham (b).

In both populations the majority of women had no problem with accessing antenatal healthcare (Figure 4). In the Birmingham population the main problem was bringing other siblings to the clinic 4/31 (13%). Women from Pretoria complained of problems with distance or cost of travel 5/31 (16%), and that there were too many people at a clinic 4/31 (13%). None of the Birmingham women stated these latter issues as problems. 

## 4. Discussion

Overall women from Birmingham attended earlier and went more often to antenatal healthcare than did women from Pretoria (Figure 2). These findings are unlikely to be a type 1 error as the sample size of this study is sufficient to provide 80% power at the 5% significance level for a comparison of the two groups using a Mann-Whitney test when the true difference in means is equal to 0.74 times the within-group standard deviation. Birmingham women had a significantly higher income, nonsignificantly higher rate of employment and had a higher level of education (Figure 1). Late attendance has been associated with educational factors by previous research [9]. Lower general education levels in women from Pretoria may explain their later use of healthcare, because they are less aware of the complications of pregnancy. This is supported by the finding that women from Pretoria could name fewer medical problems that affect pregnancy than Birmingham women. Poorer education leading to decreased knowledge of medical problems affecting pregnancy (Figure 3) may be a significant reason for poorer use of healthcare services in pregnancy in women from Pretoria. 

Similar numbers of women from Birmingham and Pretoria stated they had no problem with accessing antenatal healthcare (Figure 4). The main problem for Birmingham women was bringing another sibling, though this was more of an inconvenience than a problem blocking access to antenatal healthcare, for example, one patient said:

*“It is difficult to get to clinics when you have to bring a child that wants to be anywhere but there”* Patient 23—Birmingham interviewee.

The barriers of cost or distance to travel and clinic overcrowding (Figure 4) may help to explain why women from Pretoria access antenatal care later and make fewer visits but also demonstrates the very different problems that women from the two populations face. Provision of free transport or clinics closer to where the women live in Pretoria and child entertainment facilities in Birmingham may help tackle these problems.

Women from Birmingham stated “advice” and “reassurance” as reasons to attend antenatal healthcare appointments. If women from Birmingham stated these reasons because they gain more advice and reassurance and feel they derive benefit from appointments in this respect, they may be more willing to attend clinics than those from Pretoria. Therefore focussing antenatal healthcare in South Africa on provision of pregnancy advice and reassurance may increase antenatal healthcare use. However the ability to provide increased antenatal visits in Pretoria may be a limiting factor. 

HIV awareness is higher in women from Pretoria (Figure 3). The HIV infection rate in 2005 in the UK was 0.2% in adults aged 15–49 and 18.8% in South Africa [11]. In addition, in Johannesburg Hospital, which is less than 50 km from Pretoria the HIV prevalence is 29.4% [12]. This difference in infection rate may explain the differing concern about HIV during pregnancy, as women in South Africa are more likely to have personal or family experience of the problems of HIV/AIDS. This difference in concern about HIV exists despite both centres offering routine HIV screening and education [12, 13]. Although 21/31 (67.8%) women from Pretoria stated that HIV was a condition that could affect pregnancy, only 3/31 (9.7%) said that finding out their HIV status was an important function of antenatal healthcare. Indeed this trend is also seen in women from Birmingham where 5/31 (16.1%) stated HIV was a condition that could affect pregnancy but 0/31 (0%) felt that finding out their HIV status was a role of antenatal healthcare. This suggests that although women may be aware of the effect of HIV on pregnancy they may not see it as a risk that they identify with. The differences in perception of HIV/AIDS risk between the two regions, due partly to the difference in infection rates, but potentially also due to differences in local education policies may explain the differences in HIV/AIDS awareness. 

Research has shown that 97% of deliveries occur in NHS hospitals in the UK [2] and 83% of births are in health facilities in South Africa [8]. Indeed the study by Abrahams et al. [9] stated that the majority of South Africans give birth in healthcare facilities. Since most women in both populations give birth in health facilities, interviews carried out in the respective hospitals should be approximately representative of all births in the two populations. The Birmingham population has a diverse ethnic composition. In the UK the minority ethnic populations are concentrated in the large urban centres [14], thus the results from the Birmingham population may be representative of the general population of other large UK cities. The Pretoria population in this study is 100% black. A study by Sőderlund [15] showed that 60% of South Africans with an average per family member income of greater than R11,000 per annum (approx *£*800) use private healthcare. This study had six women with this income or above, indicating that there were approximately seven to nine patients missing from this income bracket due to the use of private healthcare. Thus the Pretoria results are at best representative of the black population who use public healthcare in large South African cities. 

All women in the Pretoria population had at least one visit to an antenatal clinic in this study. As a tertiary referral unit in South Africa, women must usually have attended one antenatal clinic in order to be referred to the Pretoria Academic hospital. Therefore these results may be an overestimation of the use of antenatal healthcare in the general Black population. 

This study showed that women use healthcare services earlier in pregnancy before the main antenatal screening tests began in the 10th–12th week [4]. This discrepancy occurs because this study recorded antenatal healthcare use as “any contact with a healthcare professional”, rather than the “first use of dedicated antenatal screening”. This study is thus concerned with antenatal healthcare not dedicated antenatal screening.

Six Birmingham women stated that they sought appointments initially to book antenatal healthcare screening, thus they were attending their GP before the start of dedicated antenatal screening. The Pretoria population first used healthcare services at a median of 16 weeks into pregnancy for the first appointment. These results were similar to the Abrahams et al. [9] study carried out in Cape Town. This indicates that urban Black populations in South Africa attend antenatal healthcare services later than urban populations in the UK. When asked why women from Pretoria booked at this time, none stated that they did so to book further healthcare and only 8/31 did so to confirm their pregnancy. This indicates that women from Pretoria found it less important to book antenatal healthcare. This lower priority for antenatal healthcare may explain their later attendance to these services.

Previous research addressing outcomes investigated in this study have indicated that reduction in number of antenatal healthcare visits is not always associated with decreased healthcare effectiveness [16]. Therefore, although this study showed a reduced number of antenatal healthcare visits by women from Pretoria, this cannot be taken as evidence of a suboptimal antenatal healthcare system in Pretoria.

## 5. Conclusions

Women from Birmingham use healthcare services earlier (*P* ≤ .0001) and more often during pregnancy (*P* ≤ .0001). Women from Birmingham identified more medical conditions that may affect pregnancy (median 6 versus 3 reasons). Women from Pretoria are more aware of HIV infection and may seek antenatal care to check their HIV status. Women from Birmingham perceived antenatal healthcare as relatively more important for advice and reassurance about pregnancy, whilst women from Pretoria had more problems with cost of transport and travel distance and clinic overcrowding. 

This study has identified a number of important differences between perceptions of antenatal healthcare in Birmingham and Pretoria, and this may help explain decreased antenatal healthcare uptake in Pretoria. Addressing lack of knowledge about medical problems in pregnancy and emphasising the importance of antenatal healthcare may increase antenatal healthcare use. In addition emphasising the aspect of advice and reassurance at antenatal healthcare clinics may help increase attendance. Problems of cost and distance of travel to clinic could be tackled by improved transport links whilst overcrowding in clinic would inevitably require increased medical staffing in Pretoria. 

A future study including perinatal or maternal morbidity and mortality would be needed to assess the effectiveness of the antenatal healthcare programs in the two locations and elucidate if reduced antenatal attendance is linked with poorer outcomes. A larger more geographically diverse population would be needed to find significance in these parameters and to confirm the findings of this study.

## Supplementary Material

The supplementary data includes the finalised structured questionnaire used in the patient interviews, the patient consent form, ethical approval from the ‘South Birmingham Research Ethics Committee' and ‘University of Pretoria' and the Birmingham Women's Hospital health care trust approval and indemnity letter.

## Figures and Tables

**Table 1:** Reasons for women seeking antenatal healthcare.

	Birmingham		Pretoria	
	*n*	(%)	*n*	(%)
Check baby health	26	83.9	26	83.9
Check own health	15	48.4	14	45.2
Advice	11	35.5	2	6.5
HIV status check	0	0	3	9.7
Felt compelled to go	3	9.7	1	3.2
Confirm pregnancy	1	3.2	2	6.5
Reassurance	5	16.1	1	3.2
Check baby orientation	0	0	3	9.7
Meet people & share experiences	2	6.5	0	0
