# Supplementary material for: An Evaluation of the Timing and Use of Healthcare during Pregnancy in Birmingham, UK and Pretoria, South Africa
Source: ISRN Obstet Gynecol. 2011 Jan 26;2011:364243. doi: 10.5402/2011/364243 (PMC3102581; doi:10.5402/2011/364243)
Supplement: Supplementary file 1 — The supplementary data includes the finalised structured questionnaire used in the patient interviews, the patient consent form, ethical approval from the ‘South Birmingham Research Ethics Committee' and ‘University of Pretoria' and the Birmingham Women's Hospital health care trust approval and indemnity letter. [file 364243.f1.pdf]

**Appendix I - Questionnaire**

A) **Socio Demographics**

1. Age __________________
2. Ethnicity

| | |
|---|---|
| White British | |
| Afro-Caribbean | |
| Asian | |
| Other | |

| | |
|---|---|
| Black South African | |
| Coloured South African | |
| White South African | |
| Other | |

3. How many years did you spend at school? ________________________________
4. What is the highest level of school you finished?

| | | | |
|---|---|---|---|
| Primary School | | | |
| Secondary School | | Technicon / College | |
| High School | | University | |

5. Do you have a job? Unemployed / Full-Time / Part-Time____________________
6. Who is the breadwinner in your household? _______________________________
7. What is you approx. monthly income? __________________________________
8. How many people live at home? ________________________________________
9. Does your household have? Electricity ☐ Water ☐
10. Where do you live? __________________________________________________

B) **Pregnancy History**

1. How old were you when you were first pregnant? __________
2. How many times have you been pregnant? ________________
3. How many children have you delivered? _________________
4. Method of delivery? Normal Vaginal ☐ C-section ☐
   Instrumental ☐

5. Who Delivered you baby? Doctor ☐ Nurse ☐ Other ☐

C) **Timing of antenatal care**

1. For your current pregnancy, when did you first go to a healthcare professional? (number of weeks / months into pregnancy)_____________________________

Why at this time?

_____________________________________________________________________________

_____________________________________________________________________________

______________________________________________________________

2. How many times did you visit a healthcare facility / have antenatal reviews?

Why at this time?

3. What do you think is the best time to first see a healthcare professional? Why?

4. What antenatal tests did you have?

| | | | | |
|---|---|---|---|---|
| U/S scan | | Amnio | | Other |
| Blood tests | | Chorion Villous Sampling | | |

D) **Reasons for antenatal care**

1. Why do you think most women seek antenatal care, in general?

2. What problems and diseases may put you or your child at risk during pregnancy?
Prompt - Infections, baby abnormalities

3. Where would you most like to have given birth? e.g. hospital / home

4. What problems do you think may occur during childbirth/delivery?

5. What are the benefits of coming to the clinic early (rather than late) in pregnancy?

6. Who else has given you advice about pregnancy (antenatal care)?

E) **Barriers to antenatal care**

1. What are the things that have made it difficult for you to see a healthcare professional / Antenatal clinics?

Healthcare Professional

| | | |
|---|---|---|
| Time | | Other |
| Distance | | |
| Cost | | |
| | | |
| | | |

2. Did you use Public / Private Antenatal Healthcare?

Public ☐ Private ☐

Why?

______________________________________________

______________________________________________

______________________________________________

______________________________________________

**Appendix II - Consent Form**

Birmingham Women's Health Care NHS

NHS Trust

Birmingham Women's Health Care NHS Trust,
Metchley Park Road,
Edgbaston,
Birmingham,
B15 2TG

Tel: 0121 472 1377

***Research Elective Project***

**An Evaluation of the timing and use of Healthcare during Pregnancy and Childbirth in Birmingham, UK, and Pretoria, South Africa.**

Dear Patient,

I am a fourth year medical student from Birmingham, United Kingdom and as part of my studies I am carrying out a short research project on the use of antenatal services (healthcare visits during pregnancy) in the UK and South Africa. Please take a few moments to decide if you would like to take part in this project. Thank you very much for taking the time to read this information

If you agree to take part you will be asked to take part in a short 20 minute interview, based on a standardised questionnaire, with myself on your use of antenatal services (healthcare personnel and hospital clinics). The questionnaire is anonymous; your name or hospital number will not be recorded. The interview is not obligatory and if you decide not to take part this will in no way affect your medical care. You may change your mind at any time during the interview and the interview will come to an end. Please sign the consent section below if you are happy to take part.

The project is being supervised by Dr Sam Pretlove and Dr H.N. Bomela.

Student ………………………………. Mark Openshaw

Supervisor……………………………. Dr H. N. Bomela / Dr S. Pretlove
*[Different for respective locations]*

**Consent**

I consent to taking part in this questionnaire.

Signed …………………………………..

Name……………………………………… Date ____________ 2007

## Appendix III - Ethical approval – South Birmingham Research Ethics Committee

**Birmingham and The Black Country** *NHS*

South Birmingham
Research Ethics Committee Strategic Health Authority
**27 Highfield Road, Edgbaston Birmingham B15 3DP**

Tel; 0121 245 2533 Fax: 0121 245 2535 ***Student Sub-Committee***

**Chairman: Mr T Marshall**

19th January 2007

Mark Openshaw
86 Gordon Road Harborne
Birmingham
B17 9EY

Dear Mr Openshaw

A COMPARISON OF PERCEPTIONS AND UTILISATION OF HEALTHCARE DURING PREGNANCY AND CHILDBIRTH IN BIRMINGHAM AND PRETORIA: S/2007/1

The student project sub-committee of the research ethics committee has reviewed your protocol and has the following comments.

1. We are sure you understand that we are not able to give permission to do thiis work in South Africa. We may be able to say that we think it is ethically acceptable, but you will have to undertake appropriate enquiries in Pretoria to ensure ethical approval there.
2. The introductory letter should be on headed paper; that of the Women's Trust is the obvious one to use.
3. There seem to be some slight incongruities in some of the questions, e.g. "Where would you most like to give birth?" Since all the subjects are post-natal, you might look at the phrasing, e.g "Where would you most like to have given biih". Other questions need looking at from a similar perspective. You may want to discuss this with your supervisor.
4. You may find that women are happy to be interviewed, but not for you to look at their medical records. It is sometimes a good idea to ask for two consents, one for the interview (consent to which could be implied) and one for looking at their records. From this perspective the only written consent you would need would be to look at the records. Again, discuss this with your supervisor.

We have no other issues regarding the project, and assuming you think again about the matters raised above, you may proceed with the project without further reference to us. You may find this approval helpful in South Africa. Please note that you will need

**Chairman: Elisabeth Buggins** *Chief Executive:* **Geoff Scaife**

permission from the R&D department of the Women's Trust before doing this work, and copies of your application and this letter have been sent to them.

Yours sincerely

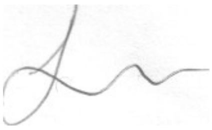

Tim Marshall

**Chairman, Student Project Sub-Committee**

**cc** **Myra** Turner

Catherine Moore Research Office, Medical
School Brian Berry, RCS University of
Birmingham
R&D department Birmingham Women's NHS Trust

## Appendix IV - Birmingham Women's Hospital Health Care Trust Approval & Indemnity

Birmingham Women's Health Care NHS
NHS Trust

06 February 2007

Mark Openshaw
86 Gordon Road
Harborne
Birmingham
B17 9EY

Dear Mark

**Re: A comparison of perceptions and utilisation of healthcare during pregnancy and childbirth in Birmingham and Pretoria**
**Student Ethics Ref: S/2007/1; R&D Approval: 06020703**

I can confirm that the R&D Department has reviewed the above project and is happy to grant Trust approval.

Your research activity is now covered by NHS indemnity as set out in HSG (96) 48, and your trial has been entered into the Trusts' database (if applicable this will be entered onto the NRR).

All research must be managed in accordance with the requirements of the Department of Health's Research Governance Framework (RGF) and to ICH-GCP standards. Your responsibilities are set out in the attached agreement.

The Trust employs the services of an external organisation to monitor 10% of all projects on an annual basis. You are contractually obliged to comply with the requests of this organisation as they have the authority to audit your site files at any time, in line with the Research Governance Framework.

If you have any queries relating to R&D, please do not hesitate to contact me. The Trust wishes you success with your research.

Yours sincerely

Julie Norris
**Project Manager**

Cc Sam Pretlove

Chairman – Ann Owen Chief Executive – Julie Burgess

Birmingham Women's Health Care NHS
NHS Trust

| | | | |
|---|---|---|---|
| **Chief Investigator:** | Mark Openshaw | **Title:** | Student |
| **Project Title:** | A comparison of perceptions and utilisation of healthcare during pregnancy and childbirth in Birmingham and Pretoria | | |
| **Sponsor:** | University of Birmingham | | |
| **Project Ref.:** | OPE001 | **R&D No:** | 06020703 |

## External Sponsorship Arrangements:

In order to comply with the EU Directive (May 04) and the Research Governance Framework, every piece of research relating to health and social care must have sponsorship arrangements.

As Sponsor for this study you have agreed to fulfil the sponsorship responsibilities.

This means that it is your responsibility to ensure:

- There is acceptable access to resources and support to deliver the proposed research.
- Responsibility is allocated for the management, monitoring and reporting of the research.
- That modification to the initial research design is approved.
- Arrangements are in place to record, report and review significant developments as the research proceeds. This includes the establishment of a data monitoring committee, monitoring of adverse events and reporting.

It is the Chief Investigator's responsibility to ensure:

- That a copy of this agreement is copied to the Sponsor if the Sponsor is not the employing organisation of the Chief Investigator
- The research proposal respects the dignity, rights, safety and well-being of participants and the relationship with care professionals.
- The research proposal is worthwhile, of high scientific quality and represents good value for money.
- The research proposal has been approved by an appropriate research ethics committee.
- Appropriate arrangements are in place for the registration of trials.
- The principal investigator, and other key researchers, have the necessary expertise and experience and have access to the resources needed to conduct the proposed research successfully.
- The arrangements and resources proposed will allow the collection of high quality, accurate data and the systems and resources being proposed are those required to allow appropriate data analysis and data protection.

- Intellectual property rights and their management are appropriately addressed in research contracts or terms of grant awards.
- Arrangements proposed for the work are consistent with the Department of Health research governance framework.
- Arrangements are in place for the sponsor and other stakeholder organisations to be alerted if significant developments occur as the study progresses, whether in relation to the safety of individuals or to scientific direction.
- An agreement has been reached about the provision of compensation in the event of non-negligent harm and any organisation, including the sponsor itself, offering such compensation has made the necessary financial arrangements.
- Arrangements are proposed for disseminating the findings.
- All scientific judgements made by the sponsor in relation to responsibilities set out here are based on independent and expert advice.
- Assistance is provided to any enquiry, audit or investigation related to the funded work.

| | | | |
|---|---|---|---|
| **Signature Chief Investigator:** | M Gpensham | **Date:** | |
| **Signed on behalf of the Trust:** | [illegible] | **Date** | 6/2/07 |
| **Position at Trust:** | Project Manage | | |

**For further queries relating to the responsibilities of the Chief Investigator, please re to section 3.8 of the Research Governance Framework or contact the R&D Office.**

Please sign and return this document to Julie Norris, c/o R&D Management Services, Birmingham Women's Hospital, Metchley Park Road, Edgbaston, Birmingham, B15 2TG.
You may wish to keep a copy for your records

## Appendix V – Ethical Approval University of Pretoria

University of Pretoria

Faculty of Health Sciences Research Ethics Committee
University of Pretoria
**Tel:** (012) 339 8619 **Fax to E-Mail:** 086 6516047
**E-Mail:** deepeka.behari@up.ac.za

Soutpansberg Road
MRC Building
Room 2 - 20

Private Bag x 385
Pretoria
0001

**Date: 25/04/2007**

| | | |
|---|---|---|
| **Number** | : | **S33/2007** |
| **Title** | : | An evaluation of the timing and use of Healthcare during Pregnancy and Childbirth in Birmingham, UK, and Pretoria, South Africa. |
| **Investigator** | : | Mark Openshaw, Department of Paediatrics, University of Pretoria (SUPERVISORS: Professor DF Wittenberg and Dr H N Bomela ) |
| **Sponsor** | : | Children's Research Fund in the United Kingdom |
| **Study Degree:** | | **Elective** (4th Year Medical Student, Birmingham University, UK) |

**This Student Protocol has been considered by the Faculty of Health Sciences Research Ethics Committee, University of Pretoria on 24/04/2007 and found to be acceptable.**

| | |
|---|---|
| Advocate AG Nienaber | (female)BA(Hons) (Wits); LLB; LLM (UP); Dipl.Datametrics (UNISA) |
| Prof V.O.L. Karusseit | MBChB; MFGP (SA); M.Med (Chir); FCS (SA): Surgeon |
| Prof M Kruger | (female) MB.ChB.(Pret); Mmed.Paed.(Pret); PhDd. (Leuven) |
| Dr N K Likibi | MB.BCh.; Med.Adviser (Gauteng Dept.of Health) |
| Dr F M Mulaudzi | (female) Department of Nursing |
| Mrs E.L. Nombe | (female) B.A. CUR Honours; MSC Nursing – UNISA (Lay Member) |
| Snr Sr J. Phatoli | (female) BCur (Et.Al) Senior Nursing-Sister |
| Dr L Schoeman | (female) Bpharm, BA Hons (Psy), PhD |
| Prof J.R. Snyman | MBChB, M.Pharm.Med: MD: Pharmacologist |
| Dr R Sommers | (female) MBChB; M.Med (Int); MPhar.Med; |
| Prof C W van Staden | MBChB; Mmed (Psych); MD; FTCL; UPLM; Dept of Psychiatry |
| Prof TJP Swart | BChD, MSc (Odont), MChD (Oral Path) Senior Specialist; Oral Pathology |
| Dr AP van der Walt | BChD, DGA (Pret) Director: Clinical Services, Pretoria Academic Hospital |

**Student Ethics Sub-Committee**

| | |
|---|---|
| Prof R S K Apatu | MBChB(Legon); PhD(Cambridge) |
| Dr A.M Bergh | (female) BA (*cum laude*), Rand Afrikaans University BA (Hons) (Linguistics), University of Stellenbosch Secondary Education Diploma (*cum laude*), University of Stellenbosch BA (Hons) (German) (*cum laude*), University of South Africa (Unisa) BEd (Curriculum Research and Non-formal Education) (*cum laude*), University of Pretoria PhD (Curriculum Studies), University of Pretoria |
| Dr S I Cronje | DD (UP) – Old Testament Theology |
| Dr M M Geyser | (female) BSc; MBChB; BSc HONS (Pharm); Dip PEC; MpraxMed |
| Mrs N Lizamore | (female) BSc(Stell), BSc (Hons) (Pret),MSc (Pret) DHETP (Pret) |
| Dr S A S Olorunju | B.Sc Hons; M.Sc; Ph.D |
| Dr L Schoeman | (female) Bpharm, BA Hons (Psy), PhD |
| Dr R Sommers | SECRETARIAT (female) MBChB; M.Med (Int); MPharMed |

**DR R SOMMERS;** MBChB; M.Med (Int); MPhar.Med.
SECRETARIAT of the Faculty of Health Sciences Research Ethics Committee
University of Pretoria

**DR L SCHOEMAN;** Bpharm, BA Hons (Psy), PhD
CHAIRPERSON of the Faculty of Health Sciences Research Students Ethics Committee – University of Pretoria

## Appendix VI - Proof of Placement in Pretoria

30 August 2006

University of Pretoria

P O Box 667 PRETORIA 0001 Republic of South Africa
Tel 012-354-2307 Fax 012-354-1754
http://www.up.ac.za/academic/medicine

Faculty of Health Sciences

Mr M R Openshaw
86 Gordon Road
Harborne
BIRMINGHAM
B17 9EY UNITED KINGDOM

Dear mr Openshaw

**ELECTIVE STUDY : DEPARTMENT OF PAEDIATRICS FROM 16.04.07 UNTIL 11.05.07**

The University of Pretoria, Faculty of Health Sciences, has accepted you as an elective student in the Department of Paediatrics for the abovementioned period. You will be working under supervision of Prof Wittenberg in the Pretoria Academic Hospital (teaching hospital).

Thank you for the elective payment of US$180. *You will be registered with the Health Professions Council of South Africa.*

Should you need transport from the Johannesburg airport to Pretoria, please contact the transport company "Wildside Tours" prior to your departure for S A. Email address: renier@wildsidetours.co.za , and telephone number: 0027 083 651 2052. They will await you at the airport with your name on a poster. The cost will be approximately R200.

Regarding accommodation, I would advise you to arrange this well in time as reasonable priced accommodation can be problematic to find in Pretoria. Sunsetview guest house (sunsetviewbb@mweb.co.za) and the student commune of dr Coert Gous are walking distance from the faculty and in the vicinity of our students who help with transport to Kalafong. His email address: cgous@tuks.co.za. You can also rent a car from him.

It is compulsory for an elective student to register as a special guest student at this University. Please report at my office, room 4-12.2, H W Snyman North Building, on the 16 April 2007 and bring your passport with you.

It is also compulsory to have adequate health and accident insurance. Please submit a letter from your insurance company with registration at my office.

As you will be a visiting student for less than three months, you have to obtain a **visitor's permit** with an endorsement that you will be visiting the University of Pretoria.

You will be most welcome at the Faculty of Health Sciences and in Pretoria.

Yours sincerely

M Reyneke

**Mrs Marie Reyneke**
**INTERNATIONAL STUDENT'S OFFICE**
